# A phase I/II trial of epirubicin and docetaxel in locally advanced breast cancer (LABC) on 2-weekly or 3-weekly schedules: NCIC CTG MA.22

**DOI:** 10.1186/s40064-015-1392-x

**Published:** 2015-10-21

**Authors:** Maureen Elizabeth Trudeau, Judith-Anne W. Chapman, Baoqing Guo, Mark J. Clemons, Rebecca A. Dent, Roberta A. Jong, Harriette J. Kahn, Kathleen I. Pritchard, Lei Han, Patti O’Brien, Lois E. Shepherd, Amadeo M. Parissenti

**Affiliations:** Sunnybrook Odette Cancer Centre, University of Toronto, 2075 Bayview Avenue, Toronto, ON M4N 3M5 Canada; NCIC Clinical Trials Group, Queen’s University, 10 Stuart Street, Kingston, ON K7L 3N6 Canada; Advanced Medical Research Institute of Canada (AMRIC), 41 Ramsey Lake Road, Sudbury, ON P3E 5J1 Canada; The Ottawa Hospital Cancer Centre, University of Ottawa, 501 Smyth Road, Ottawa, ON K1H 8L6 Canada

**Keywords:** LABC, Phase I/II, Epirubicin, Docetaxel, Microarray, Response biomarkers

## Abstract

This phase I/II neoadjuvant trial (ClinicalTrials.gov identifier NCT00066443) determined maximally-tolerated doses (MTD), dose-limiting toxicities, response-to-therapy, and explored the role of novel response biomarkers. MA.22 accrued T3N0, any N2 or N3, and T4 breast cancer patients. Treatment was 6 cycles of 3-weekly (Schedule A; N = 47) or 8 cycles of 2-weekly (Schedule B; N = 46) epirubicin/docetaxel chemotherapy in sequential phase I/II studies, with growth factor support. In phase I of each schedule, MTDs were based on DLT. In phase II, clinical responses (CR/PR) and pathologic complete responses (pCR) were assessed. Tumor biopsy cores were obtained pre-, mid-, and post-treatment: 3 for pathologic assessment; 3 for microarray studies. DLT for Schedule A was febrile neutropenia at 105 mg/m^2^ epirubicin and 75 mg/m^2^ docetaxel; for schedule B, it was fatigue at 75 mg/m^2^ for both agents. Phase II doses were 90 mg/m^2^ epirubicin/75 mg/m^2^ docetaxel for Schedule A and 60 mg/m^2^ (both agents) for Schedule B. Schedule A CR/PR and pCR rates were 90 and 10 %, with large reductions in tumor RNA content and integrity following treatment; Schedule B results were 93 and 0 %, with smaller reductions in RNA quality. Pre-treatment expression of several genes was associated with clinical response, including those within a likely amplicon at 17q12 (ERBB2, TCAP, GSDMB, and PNMT). The combination regimens had acceptable toxicity, good clinical response, induction of changes in tumor RNA content and integrity. Pre-treatment expression of particular genes was associated with clinical responses, including several near 17q12, which with ERBB2, may better identify chemoresponsiveness.

**Electronic supplementary material:**

The online version of this article (doi:10.1186/s40064-015-1392-x) contains supplementary material, which is available to authorized users.

## Background

LABC is traditionally defined as stage IIB (T3N0) and Stage IIIA/B/C from the TMN classification (Chia et al. 2008; Hortobagyi et al. 1995). LABC represents 10–15 % of breast cancer cases, with 5-year survival of 30–42 % of patients (Canadian Cancer Society et al. 2012), a significant portion of whom will have metastatic disease. However, a small subset of women who receive neoadjuvant chemotherapy and achieve a pathologic complete response (pCR), defined as no microscopic residual invasive disease following treatment, have a significantly improved 5-year disease-free survival (DFS) rate of about 87 %, and 5-year overall survival (OS) rates near 90 % (Kuerer et al. 1999). pCR rates have thus become an accepted surrogate measure for favorable long-term outcomes after neoadjuvant chemotherapy (Mieog et al. 2007; Sinclair and Swain 2010; Specht and Gralow 2009). While pCR appears to be associated with ultimate DFS and OS outcomes, patients with certain tumor subtypes have substantially lower pCR rates, but can still have favorable outcomes (Carey et al. 2007; Esserman et al. 2012; Rouzier et al. 2005). Early biomarkers, preferably before systemic therapy, would significantly enhance patient care (Carey et al. 2007; Esserman et al. 2012; Carey and Winer 2014; Cortazar et al. 2014; Houssami et al. 2012).

A number of commonly used anthracycline and taxane containing regimens have been studied in combination (TAC) or in sequence [dd(AC-P), AC-weekly P, FEC-D, AC-D] (Aigner et al. 2011; Gandhi et al. 2015; Raza et al. 2009). (Where T or D = docetaxel; P = paclitaxel; A = doxorubicin; C = cyclophosphamide; F = 5 fluorouracil; dd = dose-dense). However, optimal doses and schedules for anthracycline/taxane combination regimens have not been established for epirubicin and docetaxel. We examined the efficacies and toxicities associated with epirubicin/docetaxel combination chemotherapy at various doses and schedules for women with LABC. We also conducted exploratory tumor genome profiling studies for prospective biomarkers of response to the regimens.

## Patients and methods

### Study design

The NCIC Clinical Trials Group MA.22 is a non-randomized phase I/II clinical trial (ClinicalTrials.gov identifier NCT00066443) to determine optimal dosing regimens for docetaxel/epirubicin combination chemotherapy in women with locally advanced or inflammatory breast cancer. The protocol was approved by Health Canada and local Ethics Review Boards; patients provided written, informed consent.

Escalating doses of epirubicin and docetaxel were administered to patients in either a standard q3 weekly (Schedule A) or dose dense q2 weekly (Schedule B) regimen. Doses for Schedule A were 75 mg/m^2^ IV of docetaxel and 75, 90, 105, or 120 mg/m^2^ IV of epirubicin (with pegfilgrastim 6 mg primary prophylaxis sc per cycle on day 2). Doses for both docetaxel and epirubicin in Schedule B were 50, 60, and 70 mg/m^2^ IV (with pegfilgrastim support). For each schedule, phase I was dose finding for phase II. Cohorts of three patients were recruited at each dose level with three additional patients recruited if a dose limiting toxicity (DLT) was observed. If two or more DLTs were noted, the dose level was declared as the Maximum Tolerated Dose (MTD) and the phase II component of the trial was opened at the dose level below the MTD. DLTs may have been hematological, non-hematological, or related to the inability to deliver therapy and are defined in Table 1. None of the patients received trastuzumab initially and HER2+ patients were not enrolled on study, once trastuzumab funding became available. The microarray data have been uploaded to the Gene Expression Omnibus (GEO) portal (accession number GSE66999). This data can be found at: http://www.ncbi.nlm.nih.gov/geo/query/acc.cgi?token=irezcecwdvczvyt&acc=GSE66999.

**Table 1:** Toxicities associated with docetaxel/epirubicin chemotherapy for MA.22 patients in Schedules A or B

Toxicities^a^	Schedule A (N = 47)	Schedule B (N = 46)
Febrile neutropenia	6 (DLT Phase I)	0
Fever	1	0
Infections without neutropenia	2	1
Fatigue	1	7 (DLT Phase I)
Muscle weakness	0	1
Epistaxis	0	1
Bleeding	1	0
Pain	1	5
Cardiac LVF	0	1
Nausea/vomiting/diarrhea	6	1
Dizziness/syncope/dehydration	4	0
Neuropathy—sensory	0	3

^a^Toxicities were determined by using NCI Common Toxicity CriteriaV2.0

### Patients

Eligible patients had histologically confirmed invasive adenocarcinoma of the breast, which met the following criteria: T3N0, any N2 or N3, or inflammatory carcinoma. Patients had no clinical or radiological evidence of distant disease and no previous treatment for breast cancer. Age was >16 years, with ECOG status of 0–2, and adequate bone marrow, hepatic and renal function. Left ventricular ejection fraction must have been within the institutional norm.

### Study oversight

Data were collected, managed, and analyzed by the NCIC CTG. The NCIC CTG Data Safety Monitoring Committee reviewed study conduct, safety, and efficacy on a 6-monthly basis. The trial committee made the decision to publish the results. Manuscript writing was undertaken entirely by the first author, coauthors, and staff at the NCIC CTG central office, who vouch for the fidelity of the study and the data.

### Study endpoints

The primary objective of the phase I portion of each schedule was to identify DLTs, the MTD, and the dose for the q3 weekly and q2 weekly regimens. Phase I secondary objectives were to conduct microarray and immunohistochemical assessments of biomarker sensitivity/resistance to epirubicin and docetaxel.

The primary objective of phase II for both schedules was to evaluate clinical response rates at selected dose levels. Clinical response was defined as complete clinical response (CR; absence of disease by palpation post-treatment) or partial response (PR; patients with caliper measurements exhibiting a decrease in tumor volume ≥50 %). Patients with tumor volume decreases <50 % were considered to have stable disease (SD). If tumors increased in size, then patients had progressive disease (PD).

In phase II, secondary objectives were to examine pCR rates (pathologic absence of tumor in the breast and axillary lymph nodes at primary surgery), duration of response (time from CR or PR until progression), toxicities by NCI Common Toxicity Criteria V2.0, biomarker assessments of response to therapy, and time to distant metastasis (defined as time from registration to the first evidence of recurrent disease) and survival (time to death). The pCR rate was too low for pCR to be used as an endpoint for biomarker assessments so clinical response was used.

### Tumor core biopsies and isolation of tumor RNA

Six image-guided core biopsies of tumors were obtained pre-treatment; mid-treatment, at 3 (q3 weekly) or 4 (q2 weekly) cycles of chemotherapy; and post-treatment at 6 (q3 weekly) or 8 (q2 weekly) cycles (Additional file 1: Appendix S1). Three biopsies at each time point were stored in formalin for histological and immunohistochemical studies, while three were flash frozen and stored in liquid nitrogen (−190 °C). For RNA isolation, the methods were as previously described (Parissenti et al. 2010). Frozen biopsies were homogenized in RLT buffer and the RNA isolated using a Qiagen RNeasy™ RNA isolation kit (Qiagen Inc., Toronto, ON, Additional file 1: Appendix S1). Samples (in 35 μl of RNase-free water) were then stored at −80 °C. RNA quality was assessed by applying 1 μl of the preparation onto Caliper™ RNA nanochips (Caliper Technologies, Hopkinton, MA) and using capillary electrophoresis on an Agilent 2100 Bioanalyzer to resolve component RNAs. The Bioanalyzer software generated an RNA integrity number (RIN) between 1 and 10, where 10 represents highly intact RNA and 1 represents highly degraded RNA.

### Microarray profiling of gene expression

High RNA quality (RIN ≥5) was necessary for microarray assessment, and treatment frequently reduced mid-treatment RIN to levels <5. A surrogate endpoint of mid-treatment RIN (<5, ≥5) was utilized to examine baseline genes associated with RIN level, in addition to the examinations of genes associated with clinical response. High throughput gene expression studies using Agilent 4 × 44 k human genome arrays (27,958 human Entrez gene probes representing almost the entire protein-coding genome) were conducted on RNA preparations from pre- and mid-treatment tumor samples with RIN ≥5.0 The protocol used is described in Additional file 1: Appendix S1.

### Histological and Immunohistochemical Characterization of Tumors

The histological features as well as extent of tumor in the core biopsies were evaluated on H&E sections. Pre-treatment, the expression of cell surface receptors were assessed by immunohistochemical microscopy: estrogen receptor (ER; clone 6G11, Novocastra), progesterone receptor (PR; clone 16, Novocastra), Her2/Neu receptor (HER2; TAB 250, ZYMED), and topo2 (clone SWT3D1, DAKO) (Parissenti et al. 2010).

### Statistical analysis

Reporting is by Schedule for all analyses. All patients who received at least one dose of study treatment were included in the safety reports, subdivided by schedule and phase. Phase II DFS and OS were described with Kaplan–Meier plots. Biomarkers were assessed for all patients; ER, PR, Her2, and Topo2 immunohistochemical assessments were used to characterize tumors.

Full genome profiling was conducted using Agilent microarrays, where RNA was of sufficient quantity and quality (RIN ≥5) for profiling. Some RNAs were not available for Agilent gene profiling due to their use on prior microarrays of inferior quality, such that all patient tumors could not be profiled. The quality of the prior microarray data was insufficient to combine with the Agilent data. Exact Fisher tests (categorical factors) and Wilcoxon signed-rank tests (continuous factors) were used to compare the patient and tumor characteristics for patients with, or without, Agilent assessments. BRB array tools (version 4.2.1) were used to examine baseline gene expressions for exploratory identification of genes associated with response to epirubicin/docetaxel chemotherapy (class prediction), where response was defined in separate assessments as clinical response, and as RIN (<5, ≥5) at mid-treatment. We examined gene expression changes for patients who had both pre- and mid-treatment arrays with RIN ≥5.0 (class comparisons), to examine the association of gene expression changes with hormone receptor status (ER/PR), HER2, Topo2, and treatment dose (ANOVA to examine fixed effects; paired t-tests to examine differentially expressed genes). P values <0.001 were considered significant; we considered a false discovery rate (FDR) of <0.1.

## Results

**Fig. 1:**
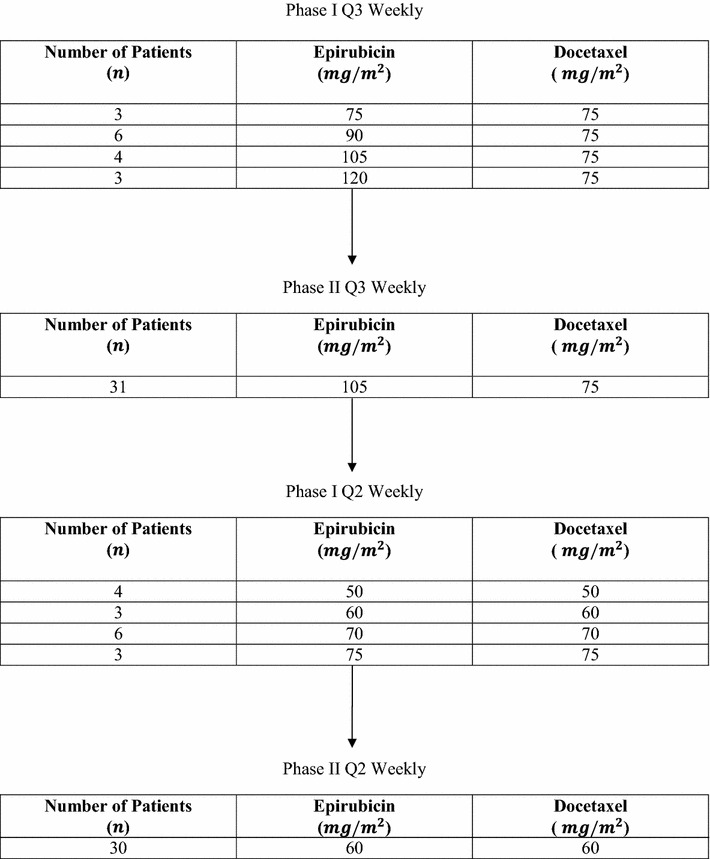
CONSORT diagram

MA.22 accrued 93 patients from February 25, 2003 to June 9, 2009; 47 to Schedule A, 46 had tumor blocks and 46 to Schedule B, all of whom had tumor blocks (CONSORT diagram, Fig. 1 outlines enrollment by schedule). Patient and tumor characteristics data are presented by schedule in Table 2. Five Schedule A and 11 Schedule B patients did not have Agilent assessments; PR levels were higher in those not assessed (p = 0.04; 0.01, respectively).

**Table 2:** Patient and tumour characteristics and response by whether patient’s tumour assessed by agilent microarray

Included phase I/II	Schedule A N with banked tumour = 46					Schedule B N with banked tumour = 46			
		Yes (N = 41)	No (N = 5)	Total (N = 46)	P value^1^	Yes (N = 35)	No (N = 11)	Total (N = 46)	P value^1^
Clinical N stage	N1	17	1	18		8	2	10	
	N2	15	3	18		24	5	29	
	N3	3	0	3		2	3	5	
	NX	6	1	7		1	0	1	
	N0	0	0	0	0.6983	0	1	1	0.1074
Clinical T stage	T2	2	0	2		1	0	1	
	T3	13	1	14		18	5	23	
	T4	26	4	30		16	4	20	
	T1	0	0	0		0	1	1	
	TX	0	0	0	1.0000	0	1	1	0.2185
Age	Median	48.5	47.8			41.8	45.4		
	Range	(27.4, 72.3)	(44.1, 72.2)		0.3061	(28.2, 65.2)	(27.5, 60.1)		0.7088
ER	Median	40	100			0	100		
	Range	(0, 100)	(0, 100)		0.3909	(0, 100)	(0, 100)		0.1919
PR	Median	0	60			0	100		
	Range	(0, 100)	(0, 100)		0.04134	(0, 100)	(0, 100)		0.01362
Her2	Median	0	0			0	0		
	Range	(0, 100)	(0, 0)		0.06909	(0, 100)	(0, 0)		0.5997
Topo2	Median	70	90			80	100		
	Range	(0, 100)	(0, 100)		0.4537	(0, 100)	(1, 90)		0.1411
% tumour extent	Median	100	90			85	80		
	Range	(40, 100)	(60, 100)		0.2862	(0, 100)	(1, 100)		0.9887

^1^P value is based on Fisher’s exact test for categorical factors and Wilcoxon signed-rank test for continuous

The phase I MTD for the three weekly regimen (Schedule A) was 120 mg/m^2^ epirubicin and 75 mg/m^2^ docetaxel, with the phase II portion conducted at 105 and 75 mg/m^2^, respectively. The clinical response rate for Schedule A was 28/31 (90.3 %), with median duration of response of 2 years (range 0.1–5.6) and 3/31 (9.7 %) pCR. For the dose dense (2 weekly) regimen (Schedule B), the MTD was 75 mg/m^2^ for both agents. In the phase 2 portion, patients received 60 mg/m^2^ of both drugs; the clinical response rate was 28/30 (93.3 %) with median duration of response of 0.2 years (range 0.1–3.9) and no pCR.

Table 1 outlines grade 3 or 4 toxicities for all patients. The DLT for Schedule A was febrile neutropenia, while for Schedule B, DLT was fatigue. None of the toxicities were unexpected for the combination of epirubicin and docetaxel.

### Disease-free survival

Recruitment for Schedule B began after that for Schedule A: median follow-up for Schedule A was 7.3 years (95 % CI 6.6–7.6), with 50 months DFS 59 % (95 % CI 39–74 %; Additional file 1: Figure S1A); Schedule B median follow-up was 3.6 years (95 % CI 3.5–4.0), with 50 months DFS 60 % (95 % CI 31–80 %; Additional file 1: Figure S1B).Time to distant mestatases and overall survival will not be reported here.

### RNA integrity (RIN) and pCR

Tumor RNA integrity decreased significantly (p < 0.001) following chemotherapy. For Schedule A, phase II patients from a pre-treatment mean of 5.4 (95 % CI 5.1–5.7) to mid-treatment 1.9 (95 % CI 1.6–2.2) and post-treatment 1.9 (95 % CI 1.5–2.3) (Additional file 1: Table S1). Mean tumor RIN values for Phase II patients in Schedule B also significantly decreased (p < 0.0001) from pretreatment 5.8 (95 % CI 5.5–6.1) to mid-treatment 4.3 (95 % CI 4.0–4.6) and post-treatment 2.9 (95 % CI 2.6–3.2). The effect of treatment on tumor RNA integrity is evident in scatter plots of maximum tumor RIN versus tumor RNA concentration for Schedule A and B patients prior to and during treatment (Additional file 1: Figure S2), where treatment resulted in a clustering of patient tumor samples to regions of low RIN and low RNA concentration values. This occurred for both patients that achieved a pCR and those that did not.

**Fig. 2:**
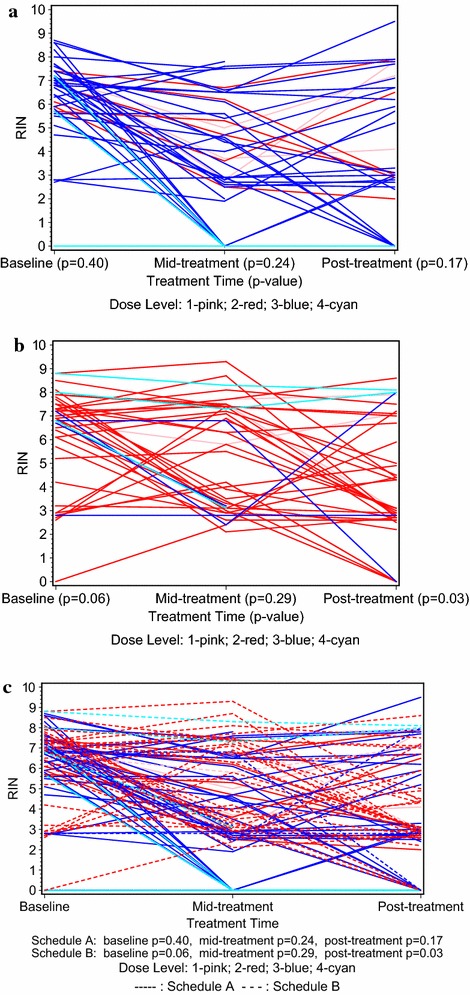
**a** Schedule A maximum RIN by dose level and treatment time. **b** Schedule B maximum RIN by dose level and treatment time. **c** Schedule A and B maximum RIN by dose level and treatment time

Figure 2 depicts maximum tumor RIN by schedule, dose level, and treatment period for all patients accrued to that schedule. Schedule A patients (Fig. 2a) did not have significantly different pre-treatment RIN, mid- and post-treatment RIN (p = 0.40; 0.24; 0.17, respectively). Schedule B patients (Fig. 2b) had different RIN pre-treatment (p = 0.06) and post-treatment (p = 0.03), although not at mid-treatment (p = 0.29). The composite of all patients accrued to two MA.22 schedules is shown in Fig. 2c and summarized in Additional file 1: Table S1.

### Analysis of gene expression in tumor core biopsies

**Fig. 3:**
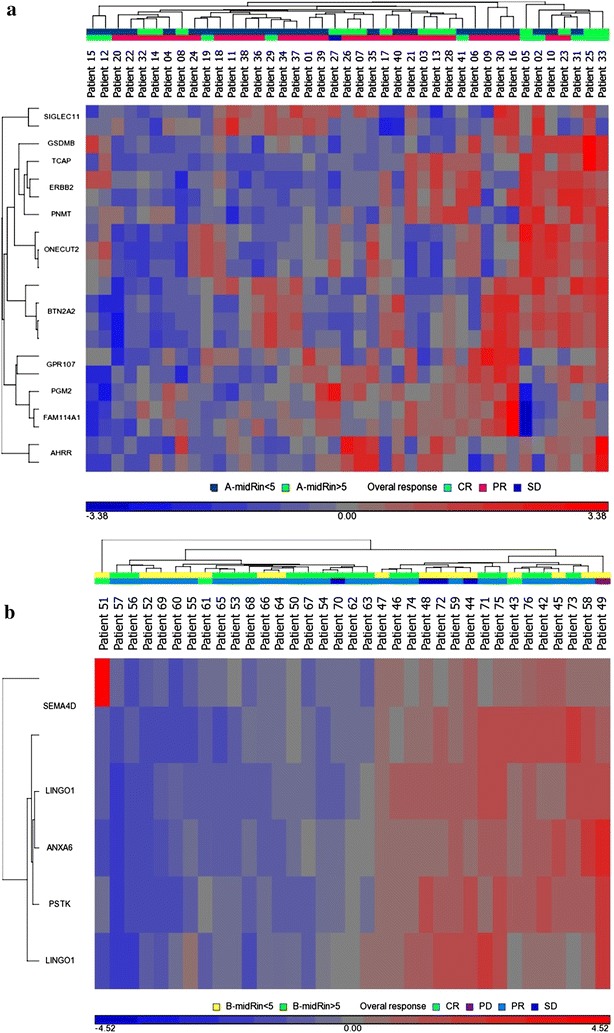
**a** Schedule A heat map of patient gene expression data. **b** Schedule B heat map of patient gene expression data

In all Schedule A patients for whom tumor RNA quality pre-treatment was sufficient in quantity and quality for DNA microarray analysis, 10 had a complete clinical response (CR), 30 had a partial response (PR), and 1 had stable disease (SD). No Schedule A patients exhibited disease progression (PD). Eleven genes had significantly altered expression in the tumors of patients who exhibited a CR in Schedule A compared to patients that had a PR, SD, or PD or were differentially expressed between 27 patients with tumor RIN <5 and 14 patients with tumor RIN ≥5 (Table 3 Schedule A for clinical response (Ammann et al. 2013; Benusiglio et al. 2006; Boucquey et al. 2006; Coon et al. 2002; Katoh 2004; Marshall et al. 1979; Nicholas et al. 2002; Pritchard et al. 2006; Qiu et al. 2014; Rauch et al. 2006; Saeki et al. 2009; Staaf et al. 2010); Additional file 1: Table S2 for RIN (Wang et al. 2011; Yosten et al. 2012; Zudaire et al. 2008); and Fig. 3a). In Schedule B, the pre-treatment expression of 9 tumor genes has associations with clinical response (3 CRs, 27 PR, 4SD, 1PD) or were differentially expressed between 18 patients with tumor RIN <5 and 17 patients with tumor RIN >5 (Table 3 Schedule B for clinical response (Biaoxue et al. 2012; Cagnoni and Tamagnone 2014; Campbell et al. 2013; Malik et al. 2014; Sakwe et al. 2011; Yuan et al. 2006; Zhang et al. 2013); Additional file 1: Table S2 for RIN (Araki and Milbrandt 2000; Feng et al. 2006; Inuzuka et al. 2005; Liedtke et al. 2007; Salzman et al. 2011); and Fig. 3b). The numbers of genes associated with change in matched pre- and mid-treatment samples and with immunohistochemical biomarkers or dose greatly exceeded the sample size so are not reported here.

**Table 3:** Genes whose expression pretreatment is associated with clinical response to chemotherapy as measured by calipers

Gene	Fold change CR/(PR + SD + PD)	Fold change CR/PR	Role and references	Chromosomal location
Schedule A				
PGM2	N.S.	−2.0	Phosphoglucomutase-2; deletion mutation in cervical cancer (Marshall et al. 1979)	4p14
GSDMB	+4.0	+3.8	Gasdermin B; overexpressed in gastric and esophageal cancers; expressed in proliferating cells, but not differentiated cells; site of recombination hotspot around ERBB2 (Katoh 2004; Saeki et al. 2009)	17q12
ERBB2	+5.6	+5.2	ERBB2; Highly correlated to clinical response in breast cancer (Coon et al. 2002; Pritchard et al. 2006)	17q12
FAM114A1	−2.0	−2.0	Also called NOXP20; protein overexpressed in the nervous system (Boucquey et al. 2006)	4p14
PNMT	+13.25		Phenylethanolamine *N*-methyltransferase; associated with ERBB2 amplification, catalyzes synthesis of epinephrine to norepinephrine (Staaf et al. 2010)	17q12
BTN2A2	+2.2		Butyrophilin; Inhibits AKT pathway; expression in epithelial and ovarian cancer associated with higher infiltrating T cells; associated with better prognosis (Ammann et al. 2013)	6p22
TCAP	+3.7		Titin-Cap protein; associated with the ERBB2 amplicon in breast cancer; associated with ERBB2 genetic polymorphisms associated with gastric cancer; associated with growth factor myostatin (Benusiglio et al. 2006; Nicholas et al. 2002; Qiu et al. 2014)	17q12
ONECUT2	+4.9		Tumor suppressor gene repressed in lung cancer; promotes growth of liver cells (Rauch et al. 2006)	18q21

Gene	Fold change CR/(PR + SD + PD)	Fold change CR/PR	Fold change PR/SD	Role and references	Chromosomal location
Schedule B					
PSTK			+2.7	Phosphoseryl tRNA Sec Kinase; involved in selenocysteine biosynthesis; important for ribosomal protein synthesis (Yuan et al. 2006)	10q
ANXA6		−1.8		Annexin A6; activates NF-ĸB survival pathway, contributes to invasiveness in breast cancer cells; correlates with poor survival/mets in lung cancer (Biaoxue et al. 2012; Campbell et al. 2013; Sakwe et al. 2011)	5q32
LINGO1			−3.1	LINGO 1 protein; promotes apoptosis (Zhang et al. 2013)	15q24
SEMA4D	+33			Semaphorin 4A; binds to Plexin B1, which physically associates with and activates ERBB2 (Cagnoni and Tamagnone 2014; Malik et al. 2014)	1q22

## Discussion

A clinical CR or PR after neoadjuvant chemotherapy are relatively common in LABC patients; however, despite these responses there is still frequently residual disease found within the breast or axilla post-treatment (Guarneri et al. 2006). While pCR rates have been associated with a survival benefit from treatment, such a benefit has not been shown for patients with LABC (Mieog et al. 2007; Sinclair and Swain 2010; Specht and Gralow 2009). Indeed, pCR rates remain low in patients with LABC, especially those patients who have ER +/Her2- disease, i.e. the majority of breast cancer patients (Kuerer et al. 1999; Guarneri et al. 2006; Liu et al. 2010). In this study, while more than 90 % of patients had either a CR or PR, less than 10 % of patients exhibited a pCR. It is likely that pCR underestimates the effects of chemotherapy in certain patients.

In this study, we provide hypothesis generating evidence for additional biomarkers that may help predict a long-term favorable response to chemotherapy or monitor response to chemotherapy in real time. Chemotherapy treatment was found to be associated with a reduction in tumor RNA content and RNA integrity.

Her2-overexpressing breast tumors are known to be highly clinically responsive to chemotherapy (Guarneri et al. 2006; von Minckwitz et al. 2012). Interestingly, for all patients undergoing microarray analysis in our study, in the absence of Herceptin, HER2*/ERBB2* transcripts were >fivefold higher in patients that had a CR after chemotherapy compared to patients with a PR, SD, or PD. The expression of 3 additional genes within 17q12 (*GSDMB*, *PNMT*, *TCAP*) had 3.7- to 13.3-fold higher expression in CR patients (Table 3 Schedule A). This higher expression may be related to an amplification of 17q12 in responding tumors. Amplification of 17q12–q21 is common in *ERBB2* overexpressing breast cancers, particularly in a 280 kb region that includes the 3 additional genes identified above (Kauraniemi and Kallioniemi 2006). Thus, in addition to *HER2/ERBB2*, our data suggests that the expression of all four genes is associated with a CR post-chemotherapy. As shown in the heat map depiction of patient gene expression data, some patients with CRs did not exhibit *HER2* overexpression, but exhibited overexpression of other genes associated with a CR, including *SINGLEC11*, *ONECUT2*, and/or *BTN2A2* (Fig. 3a).

The above overexpressed genes within 17q12 reside on chromosome 17 in the following order: *TCAP*, *PNMT*, *ERBB2*, and *GSDMB*. In this study, their tumor expression was 3.7-, 13.3-, 5.6-, and 4.0-fold higher in clinical responders, respectively. This suggests that the amplification may be centered on *PNMT* rather than *ERBB2*, or the amplification has a greater effect on *PNMT* transcription. The *PNMT* amplification or overexpression could be a more sensitive or reliable biomarker of clinically chemoresponsive breast cancer than Her2 overexpression. Moreover, the additional genes identified in this study may improve our ability to predict clinical response to chemotherapy.

The ability of these additional genes to predict or affect clinical response to chemotherapy may be related to their roles in cells. Gasdermin-B (the *GSDMB* gene product) has been shown to promote tumor formation, invasion, and metastasis in mouse xenograft models and the expression of gasdermin-related genes was correlated with reduced survival in breast cancer patients (Hergueta-Redondo et al. 2014). Thus, the poor prognosis associated with *HER2* overexpression in breast cancer (and its relationship to enhanced clinical response to chemotherapy) may also be related to the overexpression of Gasermin B. Titin-Cap (the *TCAP* gene product) blocks the secretion of myostatin, a transforming growth factor β (TGF-β) family member that negatively regulates cell growth (Nicholas et al. 2002). Since myostatin promotes apoptosis in breast tumor cells (Liu et al. 2013), the overexpression of Titin-Cap would be expected to strongly promote breast tumor proliferation. This would render cells more sensitive to killing by chemotherapy agents that target rapidly dividing tumors. It is unclear how PNMT (phenylethanolamine *N*-methyltransferase), which catalyzes the conversion of norepinephrine to epinephrine as the last step in epinephrine biosynthesis, might play a role in chemotherapy response. Two other genes whose expression pre-treatment was significantly elevated in chemoresponsive tumors (*BTN2A2* and *ONECUT2*) would be expected to improve clinical response to chemotherapy, since the enhanced expression of the former gene inhibits AKT-dependent survival pathways and is associated with improved patient prognosis (Ammann et al. 2013), while the latter gene is a known tumor suppressor gene (Rauch et al. 2006).

This study has limitations due to its small sample size; however, evidence is provided for two schedules of epirubicin and docetaxel therapy with hypothesis generating evidence for the potential value of the quality assurance measure RIN. Reductions of RIN that prevent microarray assessments after chemotherapy may in fact be a surrogate for clinical responsiveness to therapy. While most (≥90 %) of MA.22 patients had a clinical response, the proportion of patients with mid-treatment RIN that was too low for microarray assessment was much lower. However, neither clinical response nor RIN reduction was reflective of the low pCR rates seen here, which is the context in which identified genes may be useful for exploration elsewhere.

## Conclusion

In summary, the MA.22 clinical trial revealed that epirubicin/docetaxel combination chemotherapy can be delivered in LABC patients with acceptable toxicity. This regimen can, in some patients, induce strong reductions in tumor RNA integrity that are associated with post-treatment pCRs. Elevated expression of specific genes within 17q12 near the *HER2/ERBB2* locus (and other genes) were found to be associated with a complete clinical response to epirubicin/docetaxel chemotherapy. These genes could represent new targets for drug development that could improve clinical response to future regimens.

## Additional file

10.1186/s40064-015-1392-x Additional methods and clinical trial data.
