# Supplementary material for: A phase I/II trial of epirubicin and docetaxel in locally advanced breast cancer (LABC) on 2-weekly or 3-weekly schedules: NCIC CTG MA.22
Source: Springerplus. 2015 Oct 21;4:631. doi: 10.1186/s40064-015-1392-x (PMC4627986; doi:10.1186/s40064-015-1392-x)
Supplement: Supplementary file 1 — 10.1186/s40064-015-1392-x Additional methods and clinical trial data. [file 40064_2015_1392_MOESM1_ESM.docx]

**Appendix S1**

**Supplemental Methods Section**

***Isolation of image-guided needle core biopsies from patients***

The size and location of the suspicious lesion was documented using the linear array L12-5 transducer of the Philips ATL HDI 5000 SonoCT ultrasound system (Bothell, WA). After the skin was cleansed with antiseptic, the skin and breast tissue near the lesion were infiltrated with 1% lidocaine local anaesthetic and a skin nick was made with a scalpel. Under ultrasound guidance with aseptic technique, six tissue samples were obtained through the same incision using a 14 gauge needle mounted on an automated spring-loaded device. Biopsies were immediately flash-frozen and stored in liquid nitrogen (-190 ºC).

***Biopsy homogenization and RNA isolation***

To disrupt and break up cells in needle core biopsies, the frozen biopsies were placed immediately into 0.5 ml of RLT buffer (component of the Qiagen Laboratories RNeasy RNA isolation kit, Cat# 74106) in an Eppendorf tube without thawing. A sterile pellet pestle was placed onto the end of a Coreless^TM^ pellet pestle gun (both from Cosmo Bio Co. Tokyo, Japan). The biopsies were then homogenized for 5 minutes at room temperature to completely lyse the tissue. In some instances, particularly for post-treatment biopsies, samples contained significant amounts of connective tissue, which required more extensive homogenization, but did not prevent successful RNA isolation. The lysate was then passed at least 5 times through a 20-gauge needle (0.9 mm diameter) fitted to an RNase-free syringe to shear associated genomic DNA. Total RNA was then isolated from the homogenate using Qiagen RNeasy mini kits (Cat# 74106, Qiagen Laboratories, Mississauga, ON). To do this, the sample was made free of any particulate matter by centrifugation in a microfuge at maximum speed for 3 minutes, with retention of the supernatant. An equal volume of 70% ethanol was added to and mixed with the cleared lysate. The sample, including any precipitate that may have formed, was then added to an RNeasy spin column placed in a 2 ml collection tube (supplied). The sample was centrifuged for 15 s at 8000 X g, discarding the flow-through. Seven hundred 700 μl Buffer RW1 was added to the RNeasy spin column, after which the sample was washed twice in the same buffer. Bound RNA was then eluted from the column using 35 μl of RNAse-free water.

***Assessment of RNA Quality and Quantification of Extent of RNA Degradation***

RNA quality was assessed by applying 1 μl of each of the above preparations onto Caliper^TM^ RNA nanochips (Caliper Technologies, Hopkinton, MA) and resolving the various component RNAs by capillary electrophoresis on an Agilent 2100 Bioanalyzer as described in the manufacturer’s protocol: (http://www.chem.agilent.com/enUS/Search/Library‌/layouts/Agilent/PublicationSummary.aspx?whid=46787&liid=1273).

RNA standards of known mass and molecular weight were also run on the chip to determine the relative quantities of the various RNA peaks within the RNA electropherogram. Software associated with the Bioanalyzer generated an RNA integrity number (RIN) between 1 and 10 for each RNA preparation, where 10 represents highly intact RNA and 1 represented highly degraded RNA.

***Microarray Profiling of Gene Expression in Tumour Biopsies Prior to and During Treatment***

High throughput gene expression studies were conducted using pre-treatment tumour biopsies and tumour biopsies taken after 3 (Schedule A) or 4 (Schedule B) cycles of epirubicin/docetaxel chemotherapy. Agilent 4x44k human genome arrays (product number G4112F; Agilent Technologies, Mississauga, ON) were used for these studies and contain 27,958 human Entrez gene probes (close to the entire protein-coding genome). The method used was as described in the manufacturer’s protocol, which can be viewed at the following url:

<http://www.chem.agilent.com/library/usermanuals/public/g4140-90041_one-color_tecan.pdf>. Two hundred ng of total RNA from the biopsies were labelled with deoxyribonucleotide triphosphates, including a Cy3-containing deoxyribonucleosise triphosphate (Cy3-dCTP), using an Agilent Quick Amp labelling kit (Product #5190-0444). Hybridization of the labelled RNAs to the microarrays and subsequent washing of the microarrays were performed as per the manufacturer’s protocol. The microarrays were then scanned, and feature extraction and background intensity corrections performed with Agilent software (v.10.7.3.1).

**
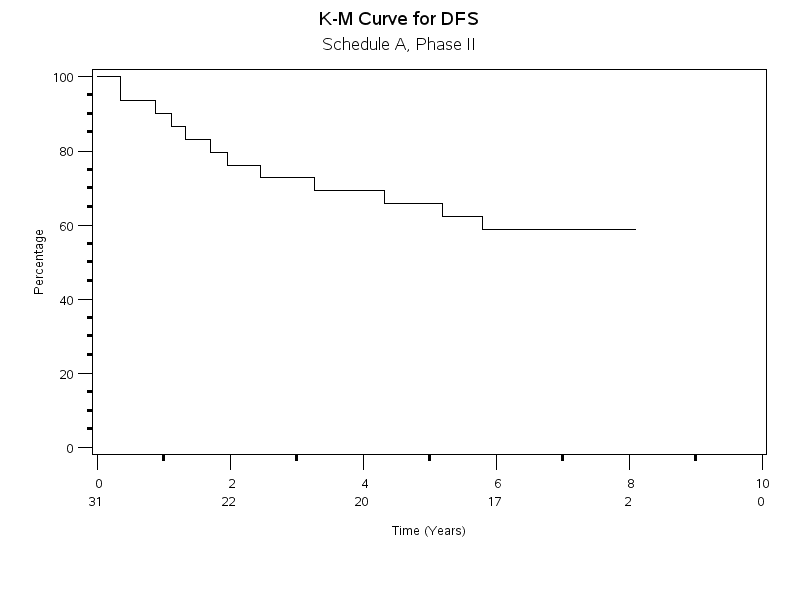
 Fig. S1A Schedule A phase II disease-free survival**

**
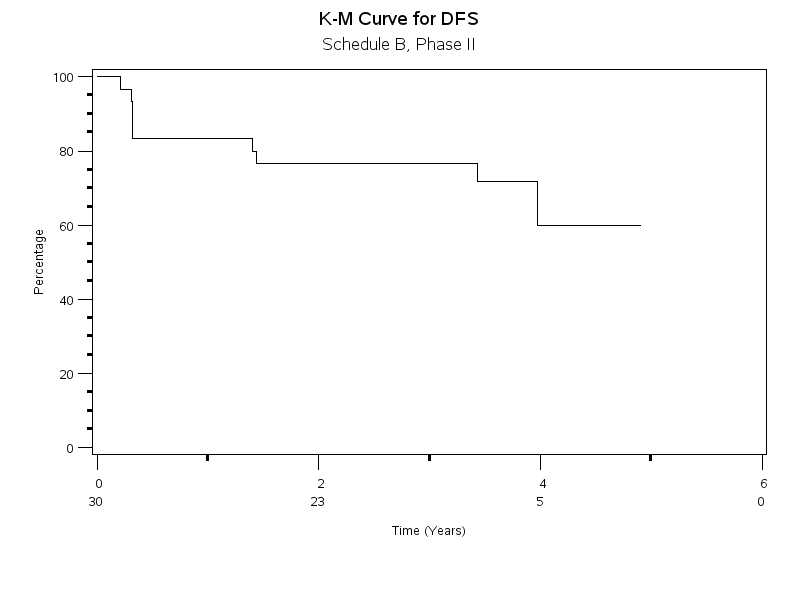
**

**Fig. S1B Schedule B phase II disease-free survival**

**
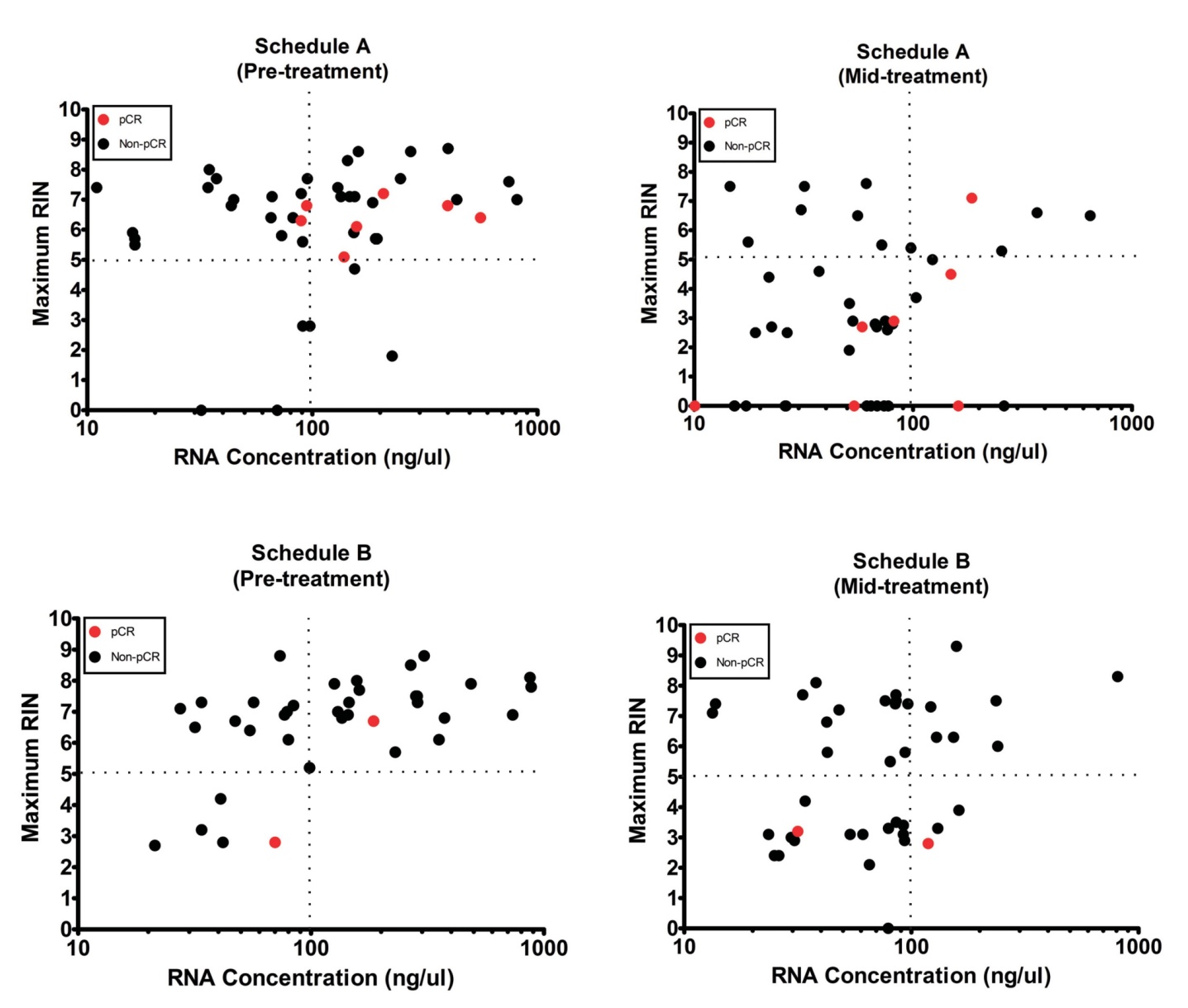
**

**Fig. S2 Differences in tumor RNA concentrations and maximum tumor RNA integrity**

**number (RIN) values between Schedule A and Schedule B patients prior to treatment**

**and at the middle of treatment. Patients that achieved a pathologic complete response**

**(pCR) and those that did not (non-pCR) are depicted in red and black symbols, respectively**

**Table S1: Effect of Treatment on Tumour RIN Values for MA.22 Patients in**

**Schedules A or B**

| **Treatment Time** | **Tumour RIN** | | **Fold Reduction in RIN Relative to**  **Pre-treatment** | | **p value**  **(Significance of Change)** | |
| --- | --- | --- | --- | --- | --- | --- |
|  | **Mean** | **Max.** | **Mean** | **Max.** | **Mean** | **Max.** |
| **Schedule A (Pre-treatment)** | 5.4 ± 0.3 | 6.2 ± 0.4 |  |  |  |  |
| **Schedule A (Mid-treatment, after 3 cycles)** | 1.9 ± 0.3 | 3.2 ± 0.5 | 2.8 | 1.9 | 0.0014 | 2.0 x 10^-6^ |
| **Schedule A (Post-treatment, after 6 cycles)** | 1.9 ± 0.4 | 2.8 ± 0.6 | 2.8 | 2.2 | 2.0 x 10^-8^ | 2.1 x 10^-6^ |
| **Schedule B (Pre-treatment)** | 5.8 ± 0.3 | 6.5 ± 0.4 |  |  |  |  |
| **Schedule B (Mid-treatment, after 4 cycles)** | 4.3 ± 0.3 | 5.1 ± 0.4 | 1.3 | 1.3 | 1.5 x 10^-13^ | 0.01 |
| **Schedule B (Post-treatment, after 8 cycles)** | 2.9 ± 0.3 | 3.7 ± 0.5 | 2.0 | 1.8 | 4.8 x 10^-12^ | 2.3 x 10^-5^ |

**Table S2: Genes Whose Expression Pretreatment is Associated with Clinical Response to Chemotherapy as Measured by Mid-Treatment Reductions in Tumor RNA Integrity**

| **Schedule A** | | | |
| --- | --- | --- | --- |
| **Gene** | **Fold Change (RIN<5/RIN≥5)** | **Role & References** | **Chromosomal Location** |
| SIGLEC11 | +1.8 | Sialic acid-binding immunoglobulin-like lectin 11; alters the secretion of growth-regulated oncogene α, IL-10, IL-7, transforming growth factor β1 and tumor necrosis factor-α (Wang et al. 2011) | 19q13 |
| GPR107 | +1.7 | G-coupled receptor; candidate receptor for neuronostatin; neuronostatin binding to GPR107 may play an important role in the control of mean arterial pressure (Yosten et al. 2012) | 9q34 |
| AHRR | -2.1 | Arylhydrocarbon Repressor; putative tumor suppressor gene; downregulation of AHRR expression in a lung cancer cell line conferred resistance to apoptotic signals and enhanced motility and invasion *in vitro* and angiogenic potential *in vivo* (Zudaire et al. 2008) | 5p15 |
| **Schedule B** | | | |
| C11ORF20 | +2.3 | Also called TEX40 (testis-expressed 40); ESRRA-C11ORF20 is a recurrent gene fusion in serous ovarian carcinoma (Salzman et al. 2011) | 11q13 |
| C10ORF125 | +2.8 | Fucose mutarotase; involved in the anomeric conversion of L-fucose. It also exhibits a pyranase activity for D-ribose (Feng et al. 2006) | 10q26 |
| CRYBB2 | +3.5 | Crystallin β-B2; promotes the growth of axons; neuronal crystallins constitute a novel class of neurite-promoting factors (Liedtke et al. 2007) | 22q11 |
| NINJ2 | +2.2 | Nerve injury-induced protein 2; upregulated in Schwann cells in the distal nerve segment after peripheral nerve injury; promotes neurite outgrowth (Araki and Milbrandt 2000) | 12p13 |
| SERINC2 | -2.6 | Serine incorporator protein 2; transmembrane proteins that facilitate incorporation of serine into phosphatidylserine and sphingolipids (Inuzuka et al. 2005) | 1p35 |
